# Dietary moderately oxidized oil activates the Nrf2 signaling pathway in the liver of pigs

**DOI:** 10.1186/1476-511X-11-31

**Published:** 2012-02-24

**Authors:** Juliane Varady, Denise K Gessner, Erika Most, Klaus Eder, Robert Ringseis

**Affiliations:** 1 Institute of Animal Nutrition and Nutrition Physiology, Justus-Liebig-University Giessen, Giessen, Germany

**Keywords:** Antioxidant enzymes, Liver, Nrf2, Oxidized fat, Phase II enzymes, Pig

## Abstract

**Background:**

Previous studies have shown that administration of oxidized oils increases gene expression and activities of various enzymes involved in xenobiotic metabolism and stress response in the liver of rats and guinea pigs. As these genes are controlled by nuclear factor erythroid-derived 2-like 2 (Nrf2), we investigated the hypothesis that feeding of oxidized fats causes an activation of that transcription factor in the liver which in turn activates the expression of antioxidant, cytoprotective and detoxifying genes.

**Methods:**

Twenty four crossbred pigs were allocated to two groups of 12 pigs each and fed nutritionally adequate diets with either fresh rapeseed oil (fresh fat group) or oxidized rapeseed oil prepared by heating at a temperature of 175°C for 72 h (oxidized fat group).

**Results:**

After 29 days of feeding, pigs of the oxidized fat group had a markedly increased nuclear concentration of the transcription factor Nrf2 and a higher activity of cellular superoxide dismutase and T4-UDP glucuronosyltransferase in liver than the fresh fat group (*P *< 0.05). In addition, transcript levels of antioxidant and phase II genes in liver, like superoxide dismutase 1, heme oxygenase 1, glutathione peroxidase 1, thioredoxin reductase 1, microsomal glutathione-S-transferase 1, UDP glucuronosyltransferase 1A1 and NAD(P)H:quinone oxidoreductase 1 in the liver were higher in the oxidized fat group than in the fresh fat group (*P *< 0.05). Moreover, pigs of the oxidized fat group had an increased hepatic nuclear concentration of the transcription factor NF-κB which is also an important transcription factor mediating cellular stress response.

**Conclusion:**

The present study shows for the first time that administration of an oxidized fat activates the Nrf2 in the liver of pigs which likely reflects an adaptive mechanism to prevent cellular oxidative damage. Activation of the NF-κB pathway might also contribute to this effect of oxidized fat.

## Background

In recent years, the contribution of oxidized fats to total energy intake has markedly increased in industrialized countries due to the rising consumption of deep-fried products [1]. In fast food restaurants, foodstuffs are typically fried in fats in fryers at temperatures close to 180°C. During the frying process, several chemical reactions occur within the frying oil resulting in the formation of a mixture of chemically distinct lipid peroxidation products [2]. Large quantities of the frying oil are absorbed into the fried food during deep-frying and therefore ingested during their consumption.

Feeding experiments with animals revealed that ingestion of oxidized fats provokes a wide array of biological effects [3-5]. One of the most striking effects of oxidized fat is the induction of oxidative stress which is due to lipid hydroperoxides absorbed from the ingested oxidized fats and reactive oxygen species (ROS) generated from microsomal cytochrome P450 enzymes which are induced by oxidized fat [6-8]. Oxidative stress in animals fed oxidized fats is evident by elevated concentrations of lipid peroxidation products, reduced concentrations of exogenous and endogenous antioxidants, and a decreased ratio of reduced and oxidized glutathione in plasma and tissues [8-13].

Previous studies have shown that administration of oxidized oils increases gene expression and activities of various enzymes involved in xenobiotic metabolism and stress response in the liver of rats and guinea pigs [14-18]. Hepatic xenobiotic metabolism and stress response is mainly controlled by nuclear factor erythroid-derived 2-like 2 (Nrf2), a master transcription factor shown to regulate more than 200 genes, including those involved in phase II detoxification and antioxidant defense [19]. Nrf2 pathway is regarded as the most important pathway in the cell to protect cells against oxidative stress [20,21]. Thus, the regulation of Nrf2 activity represents a critical step in initiating a cellular antioxidant response to ROS. It has been shown that oxidation products of n-3 fatty acids are able to activate Nrf2 in a human liver cell line, and thus to induce the expression of Nrf2 target genes involved in cellular defense [22]. More recently, we found that the ingestion of a dietary oxidized fat activates Nrf2 pathway in the intestinal mucosa of mice [23]. Based on these findings, it is likely that the induction of xenobiotic metabolism in the liver of animals fed an oxidized fat is due to an activation of Nrf2, which however has not yet been investigated. Therefore, the present study was performed to investigate the hypothesis that administration of an oxidized oil leads to an activation of Nrf2 pathway in the liver. For this end, we performed an experiment with pigs, an animal model which is closer to human physiology with respect to xenobiotic metabolism than rodents which are commonly used for the investigation of the biological effects of oxidized fats [24]. In order to reflect the practical situation of deep frying of foods in human nutrition, we used rapeseed oil--an oil commonly used for deep frying of foods--as source of fat which was heated at a temperature of 175°C for 72 h. To detect a potential activation of Nrf2 in the liver, we determined nuclear concentrations of Nrf2 and transcript levels of various Nrf2-regulated genes involved in phase II metabolism or antioxidant defense in the liver of pigs. In order to investigate whether changes in mRNA concentrations of Nrf2 target genes are reflected by altered activities, we determined activities of two Nrf2 target genes, superoxide dismutase (SOD) and thyroxine UDP-glucuronosyltransferase (T4-UGT) in the liver. As an influence on the activity of T4-UGT affects the degradation of thyroxine, we also determined the concentration of that thyroid hormone in plasma of the pigs.

## Materials and methods

### Animals and diets

For the experiment, 24 (12 male, 12 female) six week old crossbred pigs [(German Landrace × Duroc) × Pietrain] were used. The animals were kept in a pigpen controlled for temperature (23 ± 2°C), relative humidity (50-60%), and light from 06.00 to 19.00. After one week of adaptation, the pigs were weighed and randomly allocated to two groups, each consisting of 6 male and 6 female pigs, with similar average body weights. All pigs were housed in pairs in flat-deck pens and received a nutritionally adequate diet (Table 1). The diets contained 16.4 MJ metabolizable energy and 224 g crude protein per kg. The two experimental diets varied in the type of fat. The diet of the treatment group ("oxidized fat group"), contained rapeseed oil (obtained from a local supermarket) which was heated in a domestic fryer (GF-8SE, Bartscher, Salzkotten, Germany) at a temperature of 175°C for 72 h, without addition of any foodstuffs. The diet of the control group ("fresh fat group") contained a mixture of fresh rapeseed oil and fresh palm oil (91.6:8.4, w/w). This fat mixture was used in order to equalize the fatty acid composition of the two oils (since the heating process caused a partial loss of polyunsaturated fatty acids (PUFA) in the rapeseed oil). Because the frying process caused a loss of tocopherols in the rapeseed oil, the native concentrations of tocopherols of all experimental fats were analysed. Based on the native concentrations of the fats, the vitamin E concentration of the oxidized fat was adjusted to that of the fresh fat by supplementation with all-rac-α-tocopheryl acetate (the biopotency of all-rac-α-tocopheryl acetate is considered to be 67% of that of α-tocopherol). The vitamin E concentration in the fresh fat diet was 97.6 mg α-tocopherol per kg diet, and that of the oxidized fat diet was adjusted accordingly.

**Table 1:** Composition of the experimental diets

	Fresh fat diet (g/kg)	Oxidized fat diet (g/kg)
**Composition**		
Wheat	181.9	181.9
Barley	100	100
Soy bean meal (44% crude protein)	350	350
Wheat bran	146	146
Fresh rapeseed oil	137.4	-
Palm oil	12.6	-
Oxidized rapeseed oil	-	150
Choline chloride (50%)	1.5	1.5
Calcium hydrogen phosphate dihydrate	9	9
Mineral and vitamin premix*	40	40
L-Lysine (50.7%)	16.5	16.5
DL-methionine	3.55	3.55
L-threonine	3.36	3.36
L-tryptophan	0.5	0.5

*The mineral and vitamin premix provided the following per kg diet: 3.6 g lysine; 1 g methionine; 1.4 g threonine; 6.8 g calcium; 1.6 g phosphorus; 16,000 IE vitamin A; 2,000 IE vitamin D_3_; 100 mg α-tocopherol acetate; 2 g sodium; 0.8 g magnesium; 120 mg iron; 160 mg copper; 10 mg zinc; 100 mg manganese; 2 mg iodine; 0.4 mg selenium; 3 mg vitamin B1; 8 mg vitamin B2; 8 mg vitamin B6; 60 μg vitamin B12; 40 mg nicotinic acid; 20 mg pantothenic acid; 2 mg folic acid; 160 μg biotin

In order to avoid potential differences in food intake, due to adverse sensoric properties of the oxidized fat, a controlled feeding system was applied in which pigs were offered the same amount of diet during the 29 days feeding period. Water was available *ad libitum *from nipple drinkers during the entire experiment. All experimental procedures described followed established guidelines for the care and handling of laboratory animals and were approved by the local Animal Care and Use Committee (Regierungspräsidium Giessen; permission no: GI 19/3 No. 49/2010).

### Sample collection

After completion of the feeding period the animals were anaesthesised and exsanguinated 2.5 h after their last meal. Blood was collected into heparinized polyethylene tubes and plasma was subsequently obtained by centrifugation of the blood (1100 × *g*, 10 min, 4°C). Tissue samples from liver were dissected and stored at -80°C until analysis.

### Lipid analysis

The fatty acid composition of the dietary fats was determined by gas chromatography after methylation of fatty acids by trimethylsulfonium hydroxide [25]. The extent of lipid peroxidation of the experimental fats before inclusion into the diets was determined by assaying the peroxide value [26], thiobarbituric acid substances (TBARS) [26] and the percentage of total polar compounds [27].

### RNA isolation and quantitative RT-PCR (qPCR)

For the determination of mRNA expression levels total RNA was isolated from liver tissue using Trizol™ reagent (Invitrogen, Karlsruhe, Germany) according to the manufacturer's protocol. Determination of total RNA concentration and purity, cDNA synthesis and qPCR analysis were performed as described recently in detail [28]. Gene-specific primer pairs obtained from Eurofins MWG Operon were designed using Primer3 and BLAST. Features of primer pairs are listed in Table 2. For determination of relative expression levels relative quantities were calculated using GeNorm normalization factor. In order to calculate the normalization factor, all C_t_-values were transformed into relative quantification data by using the 2^-ΔCt ^equation, and the highest relative quantities for each gene were set to 1. From these values the normalization factor was calculated as the geometric mean of expression data of the three most stable out of five tested potential reference genes (Table 2). Reference gene stability across samples was determined by performing GeNorm analysis [29]. After normalization of gene expression data using the calculated GeNorm normalization factor, means and SD were calculated from normalized expression data for samples of the same treatment group. The mean of the FF group was set to 1 and mean and SD of the OF group were scaled proportionally. Data on qPCR performance for each gene measured in liver are also shown in Table 2.

**Table 2:** Characteristics and performance data of the primers used for qPCR analysis and reference gene-stability measure *M*

Gene	Forward primer (from 5' to 3') Reverse primer (from 5' to 3')	PCR product size (bp)	NCBI GenBank	Slope	R^2^	Efficiency	*M *value
*Reference genes*							
ATP5G1	CAGTCACCTTGAGCCGGGCGA TAGCGCCCCGGTGGTTTGC	94	NM_001025218.1	-0.2661	0.9981	1.85	0.036
GPI	CACGAGCACCGCTCTGACCT CCACTCCGGACACGCTTGCA	365	NM_214330.1	-0.2557	0.9964	1.80	0.034
RPS9	GTCGCAAGACTTATGTGACC AGCTTAAAGACCTGGGTCTG	327	CAA23101	-0.2705	0.9994	1.86	0.036
β-Actin	GACATCCGCAAGCACCTCTA ACATCTGCTGGAAGGTGGAC	205	NM_001167795	-0.2637	0.9979	1.84	0.046
SDHA	CTACGCCCCCGTCGCAAAGG AGTTTGCCCCCAGGCGGTTG	380	DQ402993	-0.2551	0.9986	1.80	0.041
*Target genes*							
SOD1	TCCATGTCCATCAGTTTGGA CTGCCCAAGTCATCTGGTTT	250	NM_001190422.1	-0.2773	0.9997	1.89	-
TXNR1	CTTTACCTTATTGCCCGGGT	162	NM_214154.2	-0.2615	0.9998	1.83	-
	GTTCACCGATTTTGTTGGCC						
GPX1	CTTCGAGAAGTTCCTGGTGG CCTGGACATCAGGTGTTCCT	232	NM_214201.1	-0.2594	0.9986	1.82	-
HO-1	AGCTGTTTCTGAGCCTCCAA CAAGACGGAAACACGAGACA	130	NM_001004027.1	-0.2828	0.9917	1.92	-
UGT1A1	GATCCTTTCCTGCAACGCAT GGAAGGTCATGTGATCTGAG	313	XM_001927673	-0.2395	0.9985	1.74	-
NQO1	CCAGCAGCCCGGCCAATCTG AGGTCCGACACGGCGACCTC	160	NM_001159613.1	-0.2756	0.9467	1.92	-
MGST1	TTGGCGCGCGAATCTACCACA TCCTCGGCTCCCTTCCCACTTA	239	NM_214300.1	-0.2636	0.9946	1.83	-

### Immunoblot analysis

Nuclear extracts from liver were prepared from six animals per group with a Nuclear Extract Kit (Active Motif, Rixensart, Belgium) according to the manufacturer's protocol. Protein concentrations in the nuclear extracts were determined by the bicinchoninic acid (BCA) protein assay kit (Interchim, Montluçon, France) with BSA as standard. From each sample of nuclear extract, 30 μg protein were separated on 12.5% SDS-PAGE and electro-transferred to a nitrocellulose membrane (Pall, Pensacola, FL, USA). Loading of equal amounts of protein in each line was verified by Ponceau S (Carl Roth, Karlsruhe, Germany) staining. After incubation the membranes overnight at 4°C in blocking solution, membranes were incubated with primary antibodies against Nrf2 (polyclonal anti-Nrf2 antibody; Abcam, Cambridge, UK), NF-κB/p50 (polyclonal anti-NF-κB/p50 antibody; Santa Cruz, Heidelberg, Germany) and Histone H1 (polyclonal anti-Histone H1 antibody; Active Motif, La Hulpe, Belgium) as a nuclear reference protein to control for adequate normalization at room temperature. The membranes were washed, and then incubated with a horseradish peroxidise-conjugated secondary polyclonal anti-rabbit-IgG antibody (Sigma-Aldrich, Steinheim, Germany) for Nrf2, NF-κB/p50 and Histone H1, respectively, at room temperature. Afterwards, blots were developed using ECL Advance (GE Healthcare, München, Germany). The signal intensities of specific bands were detected with a Bio-Imaging system (Syngene, Cambridge, UK) and quantified using Syngene GeneTools software (nonlinear dynamics).

### Tocopherol concentrations

Concentrations of tocopherols in experimental diets, liver and plasma were determined by high performance-liquid chromatography (HPLC) with fluorescence detection [30,31]. Tocopherol esters in the samples were saponified with sodium hydroxide. Tocopherols were extracted with n-hexane, separated isocratically on a C-18-reversed phase column (Purospher 100 RP-18; Merck-Hitachi, Darmstadt, Germany), using methanol as mobile phase and detected by fluorescence (excitation wavelength: 295 nm, emission wavelength: 325 nm).

### SOD activity

Activity of hepatic SOD was determined according to the method of Marklund and Marklund [32]. SOD activity in the homogenates was related to the protein concentration in the homogenates as determined by the BCA protein assay kit. One unit of SOD activity is defined as the amount of enzyme required to inhibit the autoxidation of pyrogallol by 50%.

### Activity of T4-UGT and plasma thyroxine concentration

The activity of T4-UGT in the liver was assayed as described by Gessner et al. [33]. In brief, hepatic microsomes were incubated in a reaction mixture containing 1 μM thyroxine (T4), 0.1 μCi of ^125^I-labeled T4, 75 mM Tris-hydrochloride (pH 7.8), 7.5 mM magnesium chloride, 5 mM UDP-glucuronic acid, 1 mM 6-propyl-2-thiouracil and 0.5 mg of microsomal protein/mL. T4 glucuronides formed during the incubation were separated from T4 and collected by HPLC. The radioactivity of the T4 glucuronide fraction as a measure of enzyme activity was counted with an automatic gamma counter (Wallac Wizard 3, Perkin Elmer, Rodgau, Germany). One unit of T4-UGT activity is defined as one pmol of T4 glucuronide formed per min. Plasma concentration of total T4 was measured with radioimmunoassay kits (MP Biomedicals, Eschwege, Germany).

### Statistical analysis

Treatment effects were analyzed with one-way ANOVA using the Minitab Statistical software Rel. 13.0 (Minitab, State college, PA, USA). Statistical significance of differences of the mean values of the two groups of pigs was evaluated using Student's *t *test. Means were considered significantly different at *P *< 0.05.

## Results

### Characterization of the experimental fats

The concentrations of the major fatty acids [palmitic acid (16:0), stearic acid (18:0), oleic acid (18:1), linoleic acid (18:2 n-6) and α-linolenic acid (18:3 n-3)] were similar between the two experimental fats (Table 3). In contrast, the oxidized fat contained higher amounts of lipid peroxidation products than the fresh fat (Table 3).

**Table 3:** Fatty acid composition and concentrations of lipid peroxidation products in the experimental fats

	Fresh fat	Oxidized fat
**Fatty acid composition (g fatty acid/100 g total fatty acids)**		
16:0	9.3	6.8
18:0	2.5	2.2
18:1	55.4	60.5
18:2 (n-6)	21.9	21.1
18:3 (n-3)	7.0	5.4
**Lipid peroxidation products**		
Polar compounds (%)	2.82	23.1
TBARS (mmol/kg)	3.24	3.39
Peroxides (mEq O_2_/kg)	0.63	7.39

### Growth performance

The food intake of the pigs was identical for both groups due to the controlled feeding system applied (Table 4). Initial body weights, daily body weight gains, feed conversion ratio and final body weights of the pigs did not differ between both groups (Table 4).

**Table 4:** Growth performance parameters and concentrations of α-tocopherol in plasma and liver of pigs fed either a fresh or an oxidized fat

	Fresh fat	Oxidized fat
**Parameters of performance**		
Initial body weight (kg)	16.5 ± 1.7	17 ± 1.3
Final body weight (kg)	40.5 ± 3.1	37.4 ± 5.2
Daily food intake (kg)	1.06 ± 0.21	1.05 ± 0.46
Daily body weight gain (g)	828 ± 135	762 ± 111
Feed conversion ratio (kg feed/kg gain)	1.31 ± 0.02	1.38 ± 0.20
**α-tocopherol concentration**		
Plasma, μmol/L	7.02 ± 1.06	3.86 ± 0.86*
Plasma, mmol/mol lipids^#^	1.84 ± 0.22	0.94 ± 0.27*
Liver, nmol/g liver	19.2 ± 2.79	11.9 ± 2.98*
Liver, mmol/mol lipids^#^	0.144 ± 0.07	0.087 ± 0.03*

Results are shown as mean ± SD (n = 12/group). *Means are significantly different, *P *< 0.05. ^#^Sum of triglycerides and cholesterol

### Tocopherol concentrations in plasma and liver

In order to assess effects of the oxidized fat on the antioxidative status of the animals, we determined concentrations of tocopherol in plasma and liver. Concentrations of α-tocopherol in plasma and liver - both, on absolute terms and expressed per mol of lipids--were reduced in the oxidized fat group compared to the fresh fat group (*P *< 0.05; Table 4). Concentrations of other tocopherols, beside α-tocopherol, in plasma and liver were negligible in both groups.

### Nuclear concentration of Nrf2 in the liver

**Figure 1:**
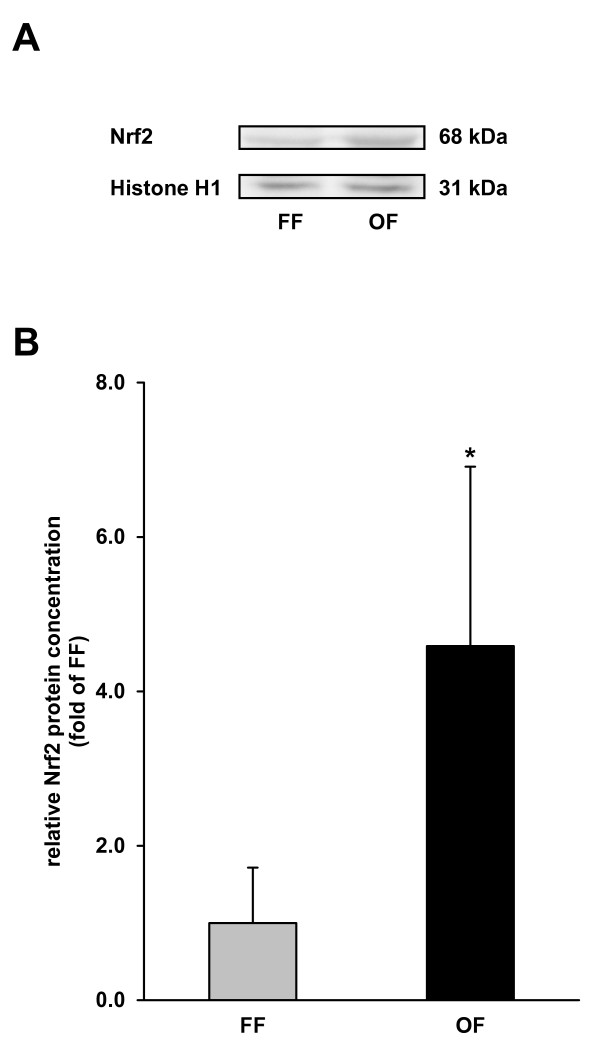
**Nuclear concentration of Nrf2 in the liver of pigs fed either a fresh fat or an oxidized fat**. (**A**) Representative immunoblots specific to Nrf2 and Histone H1 for normalization are shown for one sample per group. Immunoblots for the other samples revealed similar results. (**B**) Bars represent data from densitometric analysis and represent means ± SD (n = 6/group). Bars represent fold of relative protein concentration of the fresh fat group. *Different from pigs fed the fresh fat diet, *P *< 0.05. FF, fresh fat group; OF, oxidized fat group.

Activation of Nrf2 causes a translocation from cytosol into the nucleus. Therefore, the concentration of Nrf2 in the nuclear fraction was determined to evaluate activation of Nrf2 by the oxidized fat. Pigs receiving the oxidized fat had 4.6-fold higher nuclear concentrations of Nrf2 in the liver than pigs receiving the fresh fat (*P *< 0.05; Figure 1).

### Relative mRNA concentration of Nrf2-regulated genes in the liver

**Figure 2:**
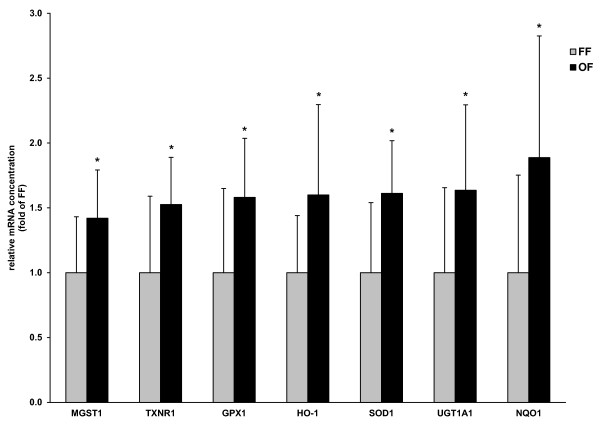
**Relative mRNA concentrations of MGST1, TXNR1, GPX1, HO-1, SOD1, UGT1A1 and NQO1 in liver of pigs fed either a fresh fat or an oxidized fat**. Bars represent mean ± SD (n = 12/group), and are expressed as fold of relative mRNA concentration of the fresh fat group. *Different from pigs fed the fresh fat, *P *< 0.05. FF, fresh fat group; GPX1, glutathione peroxidase 1; HO-1, heme oxygenase 1; MGST1, microsomal glutathione-S-transferase 1; NQO1, NAD(P)H:quinone oxidoreductase 1; OF, oxidized fat group; SOD1, Co/Zn-superoxide dismutase; TXNR1, thioredoxin reductase 1; UGT1A1, UDP glucuronosyltransferase 1A1.

Pigs fed the oxidized fat had higher relative mRNA concentrations of microsomal glutathione-S-transferase 1 (MGST1), Co/Zn-superoxide dismutase (SOD1), thioredoxin reductase 1 (TXNR1), glutathione peroxidase 1 (GPX1), heme oxygenase 1 (HO-1), NAD(P)H:quinone oxidoreductase 1 (NQO1) and UDP glucuronosyltransferase 1A1 (UGT1A1) in the liver than pigs of the fresh fat group (*P *< 0.05; Figure 2).

### Nuclear concentration of NF-κB in the liver

**Figure 3:**
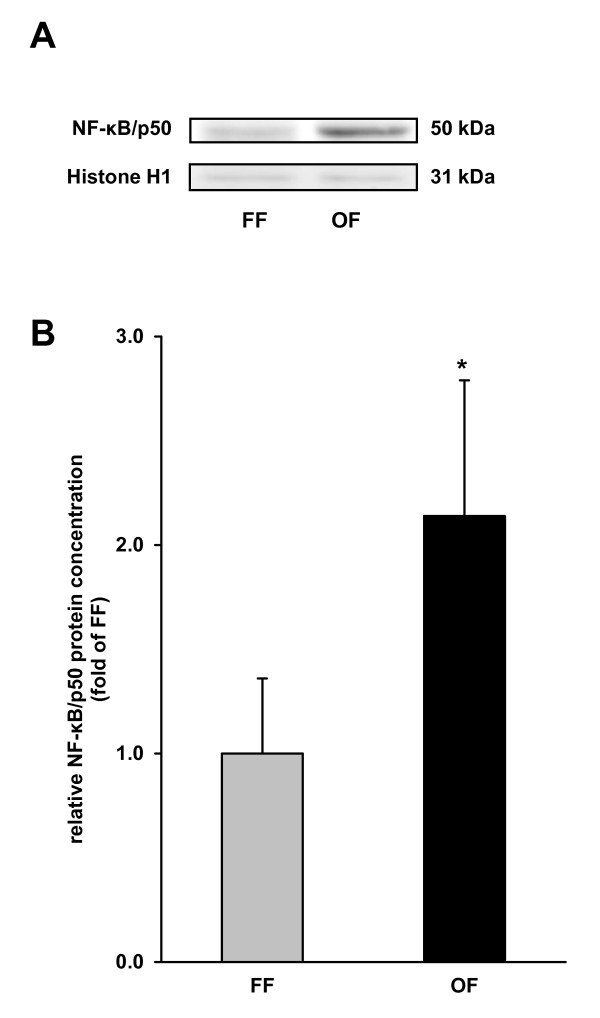
**Nuclear concentration of NF-κB/p50 in the liver of pigs fed either a fresh fat or an oxidized fat**. (**A**) Representative immunoblots specific to NF-κB/p50 and Histone H1 for normalization are shown for one sample per group. Immunoblots for the other samples revealed similar results. (**B**) Bars represent data from densitometric analysis and represent means ± SD (n = 6/group). Bars represent fold of relative protein concentration of the fresh fat group. *Different from pigs fed the fresh fat diet, *P *< 0.05. FF, fresh fat group; OF, oxidized fat group.

To further study whether other oxidative stress-sensitive transcription factors than Nrf2 are activated by the oxidized fat, we determined the concentration of the p50 subunit of NF-κB in the nuclear fraction of the liver homogenates. Pigs receiving the oxidized fat had 2.2-fold higher nuclear concentrations of NF-κB/p50 in the liver than pigs receiving the fresh fat (*P *< 0.05; Figure 3).

### Activities of SOD and T4-UGT in the liver

Pigs fed the oxidized fat had higher activities of SOD and T4-UGT in the liver than pigs fed the fresh fat (SOD: oxidized fat group, 105 ± 25 U/mg protein; fresh fat group, 75 ± 36 U/mg protein; T4-UGT: oxidized fat group, 4.4 ± 0.4 U/mg protein; fresh fat group, 3.8 ± 0.3 U/mg protein; *P *< 0.05 for both enzymes).

### Concentration of thyroxine in plasma

In order to assess whether an increased activity of T4-UGT in the liver was leading to an alteration of thyroxine status, we determined the concentration of total thyroxine in plasma which was lower in pigs fed the oxidized fat than in pigs fed the fresh fat (oxidized fat group: 57.9 ± 14.6 nmol/L; fresh fat group: 72.7 ± 16.9 nmol/L; *P *< 0.05).

## Discussion

The aim of this study was to investigate the hypothesis that consumption of a dietary oxidized fat leads to an activation of Nrf2 in the liver which in turn induces expression of genes involved in antioxidant defense and phase II metabolism. As a source of oxidized fat, we used rapeseed oil heated in a domestic fryer under practical conditions. Rapeseed oil is widely used for frying of foods in households and restaurants due to some advantages of partially hydrogenated fats. First, it has a more favourable fatty acid composition with respect to human health, particularly a favourable balance of n-3 to n-6 PUFA and a lower saturated fatty acid content. Second, it has the advantage that is must not be melted prior to use.

Critical evaluation of the concentrations of lipid peroxidation products (peroxides, TBARS, polar compounds) indicates that oxidation of PUFA in the oil during heating occurred. Nevertheless, concentrations of these lipid peroxidation products were lower than in most other studies dealing with the effects of oxidized fats [34-36]. Although the parameters determined to assess the degree of oxidation such as TBARS are not very specific, the oil used in this study can be regarded as moderately oxidized. On the base of analyses of a large number of used frying fats, it has been found that heated fats are acceptable until they reach a level of 25% polar compounds [37]. Thus, the oxidized fat used in this study, containing 23% of polar compounds, would be just acceptable for frying of foods.

Heating an oil leads to a loss of its PUFA and its native antioxidants such as tocopherols [2,38]. In order to avoid secondary effects on metabolism due to differences in the intake of PUFA and antioxidants between the two groups, we equalized the control fat and the oxidized fat for their fatty acid compositions and their vitamin E concentrations. The vitamin E concentrations of the diets, being around 90 mg α-tocopherol equivalents per kg diet, were in excess of the vitamin E requirement which might be around 40 mg α-tocopherol equivalents per kg for the specific diets used in this study (according to calculations of the vitamin E requirement as a function of amount and type of PUFA as suggested by Muggli [39]).

In most studies dealing with the effects of thermoxidized fats, feeding the diet containing the oxidized fat lowered body weight gains in the experimental animals due to a diminished feed intake, a reduced digestibility of nutrients and general toxic effects [34,35,40-42]. In the present study, feeding the diet containing the oxidized fat did not influence the growth of the pigs. This might be due to the facts that feed intake was equalized between the two groups of pigs by a controlled feeding system and that the heated oil was only moderately oxidized. The finding that the body weight development was not affected by the oxidized fat is advantageous from a methodological viewpoint because the effects of the oxidized fat were not confounded by secondary effects of reduced growth. Recent studies have shown that consumption of oxidized fats leads to a reduction of tocopherol concentrations in animal tissues due to a reduced digestibility and an enhanced turnover of vitamin E [8,11,12,43]. The finding of reduced α-tocopherol concentrations in plasma and liver of pigs fed the oxidized fat in the present study indicates that even moderately oxidized fats compromise tissue vitamin E status in animals.

According to the hypothesis of this study, we observed for the first time that administration of an oxidized fat leads to an activation of the transcription factor Nrf2 in the liver. Activation of Nrf2 was evident by an increased concentration of nuclear Nrf2 and increased transcript levels of several Nrf2 target genes involved in the antioxidant defense system (SOD1, GPX1, TXNR1, HO-1) and phase II metabolism (NQO1, MGST1, UGT1A1). Under normal conditions, Nrf2 is located in the cytosol of cells where it is constantly degraded via the proteasomal system. In response to various stimuli like oxidative stress, Keap1--a cytosolic inhibitory protein--dissociates from Nrf2 and Nrf2 translocates into the nucleus, where it binds to specific DNA-sequences called antioxidant response element (ARE) in the regulatory region of target genes [44]. Regarding that Nrf2 is activated by ROS [45,46], the occurrence of oxidative stress commonly observed in animals fed oxidized fats might be one possible explanation for activation of Nrf2 in pigs fed the oxidized fat. This possibility is strengthened by the observation that the up-regulation of enzymes involved in xenobiotic metabolism and stress response due to feeding of oxidized fats was attenuated by concomitant supplementation of vitamin C or vitamin E in guinea pigs and rats, resp [15,16]. Recently, it has been found that n-3 fatty acid oxidation products deriving from eicosapentaenoic or docosahexaenoic acid are able to directly activate Nrf2 by initiating dissociation of Keap1 [22]. Therefore, the possibility that Nrf2 activation was directly induced by specific oxidation products present in the oxidized oil cannot be excluded.

Some of the genes determined in this study, such as HO-1, GPX1 and SOD1, also contain binding sites for other oxidative stress-sensitive transcription factors, such as NF-κB, in their regulatory region [47-49]. Therefore, it is likely that the up-regulation of those genes was at least in part mediated by activation of NF-κB observed in the pigs fed the oxidized fat, as indicated by an increased nuclear concentration of p50. Thus, it is very likely that a coordinated activation of multiple oxidative stress-sensitive signaling pathways is responsible for the observed up-regulation of antioxidant, cytoprotective and detoxifying genes in the liver of pigs fed the oxidized fat diet, particularly because ROS, whose formation is induced by the administration of oxidized fats, are common stimuli of these pathways.

A further interesting observation of this study is that hepatic activity of T4-UGT, an enzyme which belongs to the phase II system, in the liver was increased in pigs fed the oxidized fat. T4-UGT catalyses the glucuronidation of thyroxine, and thus is the key enzyme of its elimination in the liver [50]. The finding of an increased activity of that enzyme concurs with a reduced concentration of total thyroxine in plasma of pigs fed the oxidized fat suggesting that hepatic elimination of that hormone was enhanced in those pigs. Thus, the present study indicates that oxidized fats could affect thyroid hormone status via an induction of the thyroid hormone elimination process in the liver such as observed for several other compounds which are acting as inducers of the hepatic xenobiotic system [51-53]. It is well known that hepatic phase I and phase II enzymes play an important role in elimination and detoxification of many drugs [54,55]. Thus, it is likely that ingestion of oxidized fats enhances the elimination of medical drugs, due to an induction of the hepatic xenobiotic system.

In conclusion, the present study shows for the first time that the ingestion of a moderately oxidized oil causes an activation of Nrf2 in the liver of pigs. This probably provides an explanation for the concomitant up-regulation of hepatic genes involved in the antioxidant defense system and phase II metabolism as observed herein in pigs and in recent studies in rats and guinea pigs [14-19]. Induction of Nrf2 in the liver of pigs fed an oxidized fat can be interpreted as an adaptive response of the liver to cope with oxidative stress induced by administration of oxidized fats, thereby, preventing ROS-mediated damage. Activation of Nrf2 is generally regarded as a beneficial effect as it causes an up-regulation of a wide spectrum of antioxidant, cytoprotective and detoxifying genes and thus protects the cell against ROS and toxic compounds. Indeed, the coordinated up-regulation of these genes by Nrf2 activators is considered as a potential therapeutic strategy to protect against insults such as inflammation and oxidative stress induced in various chronic diseases [56]. Nevertheless, the results of the present study must not be interpreted in the way that oxidized fats can be regarded as health-promoting components of the diet, as components of oxidized fats might have several adverse effects in human subjects. The results of this study rather suggest that oxidized fats are a mixture of chemically distinct substances, some of which exhibit a significant biological activity.

## Abbreviations

FF: Fresh fat; GPX1: Glutathione peroxidase 1; HO-1: Heme oxygenase 1; Keap1: Kelch-like ECH-associated protein 1; MGST1: Microsomal glutathione-S-transferase 1; NQO1: NAD(P)H: Quinone oxidoreductase 1; OF: Oxidized fat; PUFA: Polyunsaturated fatty acid; SOD1: Co/Zn-superoxide dismutase; TBARS: Thiobarbituric acid substances; T4-UGT: Thyroxine UDP-glucuronosyltransferase; TXNR1: Thioredoxin reductase 1; UGT1A1: UDP Glucuronosyltransferase 1A1.

## Competing interests

The authors declare that they have no competing interests.

## Authors' contributions

JV carried out the experiments and participated in the interpretation of the data and drafted the manuscript. DKG and EM participated in analysis. KE and RR conceived of the study and its design, coordinated work, participated in the interpretation of the results, and helped to draft the manuscript. All authors read and approved the final manuscript.

## References

[B1] Guthrie JF Lin BH Frazao E Role of food prepared away from home in the American diet, 1977-78 versus 1994-96: changes and consequences J Nutr Educ Behav 2002 34 140 150 10.1016/S1499-4046(06)60083-3 12047838

[B2] Choe E Min DB Chemistry of deep-fat frying oils J Food Sci 2007 72 77 86 10.1111/j.1750-3841.2007.00352.x 17995742

[B3] Cohn JS Oxidized fat in the diet, postprandial lipaemia and cardiovascular disease Curr Opin Lipidol 2002 13 19 24 10.1097/00041433-200202000-00004 11790959

[B4] Staprans I Pan XM Rapp JH Feingold KR The role of dietary oxidized cholesterol and oxidized fatty acids in the development of atherosclerosis Mol Nutr Food Res 2005 49 1075 1082 10.1002/mnfr.200500063 16270280

[B5] Ringseis R Eder K Regulation of genes involved in lipid metabolism by dietary oxidized fat Mol Nutr Food Res 2011 1 109 121 10.1002/mnfr.201000424 21207516

[B6] Staprans I Rapp JH Pan XM Kim KY Feingold KR Oxidized lipids in the diet are a source of oxidized lipid in chylomicrons of human serum Arterioscler Thromb 1994 14 1900 1905 10.1161/01.ATV.14.12.1900 7981177

[B7] Hayam I Cogan U Mokady S Dietary oxidized oil and the activity of antioxidant enzymes and lipoprotein oxidation in rats Nutr Res 1995 15 1037 1044 10.1016/0271-5317(95)00065-Q

[B8] Keller U Brandsch C Eder K Supplementation of vitamins C and E increases the vitamin E status but does not prevent the formation of oxysterols in the liver of guinea pigs fed an oxidised fat Eur J Nutr 2004 43 353 359 10.1007/s00394-004-0481-3 15309456

[B9] Izaki Y Yoshikawa S Uchiyama M Effect of ingestion of thermally oxidized frying oil on peroxidative criteria in rats Lipids 1984 19 324 331 10.1007/BF02534782 6738310

[B10] Kok TS Harris PG Alexander JC Heated canola oil and oxidative stress in rats Nutr Res 1988 8 673 684 10.1016/S0271-5317(05)80081-8

[B11] Liu JF Huang CJ Tissue alpha-tocopherol retention in male rats is compromised by feeding diets containing oxidized frying oil J Nutr 1995 125 3071 3080 7500186 10.1093/jn/125.12.3071

[B12] Liu JF Huang CJ Dietary oxidized frying oil enhances tissue alpha-tocopherol depletion and radioisotope tracer excretion in vitamin E-deficient rats J Nutr 1996 126 2227 2235 8814211 10.1093/jn/126.9.2227

[B13] Eder K Keller U Hirche F Brandsch C Thermally oxidized dietary fats increase the susceptibility of rat LDL to lipid peroxidation but not their uptake by macrophages J Nutr 2003 133 2830 2837 12949373 10.1093/jn/133.9.2830

[B14] Huang CJ Cheung NS Lu VR Effects of deteriorated frying oil and dietary protein levels on liver microsomal enzymes in rats J Am Oil Chem Soc 1988 65 1796 1803 10.1007/BF02542385

[B15] Liu JF Chan FC Forms of cytochrome P450 in the liver microsome of oxidized frying oil-fed guinea pigs J Nutr Sci Vitaminol 2000 46 240 245 10.3177/jnsv.46.240 11234917

[B16] Sülzle A Hirche F Eder K Thermally oxidized dietary fat upregulates the expression of target genes of PPARα in rat liver J Nutr 2004 134 1375 1383 15173399 10.1093/jn/134.6.1375

[B17] Chen YY Chen CM Chao PY Chang TJ Liu JF Effects of frying oil and Houttuynia cordata thunb on xenobiotic-metabolizing enzyme system of rodents World J Gastroenterol 2005 11 389 392 15637750 10.3748/wjg.v11.i3.389 PMC4205344

[B18] Liu JF Lee YW Chang FC Effect of oxidized frying oil and vitamin C levels on the hepatic xenobiotic-metabolizing enzyme system of guinea pigs J Nutr Sci Vitaminol 2000 46 137 140 10.3177/jnsv.46.137 10955280

[B19] Kwak MK Wakabayashi N Itoh K Motohashi H Yamamoto M Kensler TW Modulation of gene expression by cancer chemopreventive dithiolethiones through the Keap1-Nrf2 pathway. Identification of novel gene clusters for cell survival J Biol Chem 2003 278 8135 8145 10.1074/jbc.M211898200 12506115

[B20] Jaiswal AK Nrf2 signaling in coordinated activation of antioxidant gene expression Free Radic Biol Med 2004 36 1199 1207 10.1016/j.freeradbiomed.2004.02.074 15110384

[B21] Copple IM Goldring CE Kitteringham NR Park BK The Nrf2-Keap1 defence pathway: role in protection against drug-induced toxicity Toxicol 2008 246 24 33 10.1016/j.tox.2007.10.029 18083283

[B22] Gao L Wang J Sekhar KR Yin H Yared NF Schneider SN Sasi S Dalton TP Anderson ME Chan JY Morrow JD Freeman ML Novel n-3 fatty acid oxidation products activate Nrf2 by destabilizing the association between Keap1 and Cullin3 J Biol Chem 2007 282 2529 2537 17127771 10.1074/jbc.M607622200

[B23] Varady J Eder K Ringseis R Dietary oxidized fat activates the oxidative stress-responsive transcription factors NF-κB and Nrf2 in intestinal mucosa of mice Eur J Nutr 2011 50 601 609 10.1007/s00394-011-0181-8 21544513

[B24] Puccinelli E Gervasi PG Longo V Xenobiotic metabolizing cytochrome P450 in pig, a promising animal model Curr Drug Metab 2011 12 507 525 21476973 10.2174/138920011795713698

[B25] Butte W Rapid method for the determination of fatty acid profiles from fats and oils using trimethylsulfonium hydroxide for transesterification J Chromatogr 1983 261 142 145

[B26] Deutsche Gesellschaft für Fettwissenschaft Einheitsmethoden zur Untersuchung von Fetten, Fettprodukten, Tensiden und verwandten Stoffen 1994 Stuttgart: Wissenschaftliche Verlagsgesellschaft

[B27] International Union of Pure and Applied Chemistry (IUPAC) Determination of polar compounds, polymerized and oxidized triacylglycerols, and diacylglycerols in oils and fats Pure Appl Chem 2000 72 1563 1575 10.1351/pac200072081563

[B28] Keller J Ringseis R Koc A Lukas I Kluge H Eder K Supplementation with L-carnitine downregulates genes of the ubiquitin proteasome system in the skeletal muscle and liver of piglets Animal 2011 6 70 78 10.1017/S1751731111001327 22436156

[B29] Vandesompele J De Preter K Pattyn F Poppe B Van Roy N De Paepe A Speleman F Accurate normalization of real-time quantitative RT-PCR data by geometric averaging of multiple internal control genes Genome Biol 2002 3 RESEARCH0034 12184808 10.1186/gb-2002-3-7-research0034 PMC126239

[B30] Catignani GL Bieri JG Simultaneous determination of retinol and alpha-tocopherol in serum or plasma by liquid chromatography Clin Chem 1983 29 708 712 6831704

[B31] Cort WMS Vicente TS Waysek EH Williams BD Vitamin E content of feedstuffs determined by high-performance liquid chromatographic fluorescence J Agric Food Chem 1983 31 1330 1333 10.1021/jf00120a045 6689171

[B32] Marklund S Marklund G Involvement of the superoxide anion radical in the autoxidation of pyrogallol and a convenient assay for superoxide dismutase Eur J Biochem 1974 47 469 474 10.1111/j.1432-1033.1974.tb03714.x 4215654

[B33] Gessner DK Ringseis R Möller C Eder K Increased plasma thyroid hormone concentrations in LDL receptor deficient mice may be explained by inhibition of aryl hydrocarbon receptor-dependent expression of hepatic UDP-glucuronosyltransferases Biochim Biophys Acta in press 10.1016/j.bbagen.2011.12.003 22185956

[B34] Yoshida H Kajimoto G Effect of dietary vitamin E on the toxicity of autoxidized oil to rats Ann Nutr Metab 1989 33 153 161 10.1159/000177532 2802528

[B35] Corcos Benedetti P Di Felice M Gentili V Tagliamonte B Tomassi G Influence of dietary thermally oxidized soybean oil on the oxidative status of rats of different ages Ann Nutr Metab 1990 34 221 231 10.1159/000177591 2400204

[B36] Ammouche A Rouaki F Bitam A Bellal MM Effect of ingestion of thermally oxidized sunflower oil on the fatty acid composition and antioxidant enzymes of rat liver and brain in development Ann Nutr Metab 2002 46 268 275 10.1159/000066496 12464727

[B37] Frankel EN Lipid oxidation 1998 Dundee, UK: The Oily Press

[B38] Eder K Kirchgessner M The effect of dietary vitamin E supply and a moderately oxidized oil on activities of hepatic lipogenic enzymes in rats Lipids 1998 33 277 283 10.1007/s11745-998-0206-x 9560802

[B39] Muggli R Physiological requirements of vitamin E as a function of the amount and type of polyunsaturated fatty acid World Rev Nutr Diet 1994 75 166 168 7871821 10.1159/000423574

[B40] Blanc P Revol A Pacheco H Chronical ingestion of oxidized oil in the rat: effect on lipid composition and cytidilyl transferase activity in various tissues Nutr Res 1992 12 833 844 10.1016/S0271-5317(05)80641-4

[B41] Hochgraf E Mokady S Cogan U Dietary oxidized linoleic acid modifies lipid composition of rat liver microsomes and increases their fluidity J Nutr 1997 127 681 686 9164986 10.1093/jn/127.5.681

[B42] Engberg RM Lauridsen C Jensen SK Jakobsen K Inclusion of oxidized vegetable oil in broiler diets. Its influence on nutrient balance and on the antioxidative status of broilers Poult Sci 1996 75 1003 1011 8829233 10.3382/ps.0751003

[B43] Eder K Stangl GI Plasma thyroxine and cholesterol concentrations of miniature pigs are influenced by thermally oxidized dietary lipids J Nutr 2000 130 116 121 10613777 10.1093/jn/130.1.116

[B44] Dhakshinamoorthy S Long DJ Jaiswal AK Antioxidant regulation of genes encoding enzymes that detoxify xenobiotics and carcinogens Curr Top Cell Regul 2000 36 201 216 10842753 10.1016/s0070-2137(01)80009-1

[B45] Kaspar JW Niture SK Jaiswal AK Nrf2:INrf2 (Keap1) signaling in oxidative stress Free Radic Biol Med 2009 47 1304 1309 10.1016/j.freeradbiomed.2009.07.035 19666107 PMC2763938

[B46] Niture SK Kaspar JW Shen J Jaiswal AK Nrf2 signaling and cell survival Toxicol Appl Pharmacol 2010 244 37 42 10.1016/j.taap.2009.06.009 19538984 PMC2837794

[B47] Lavrovsky Y Schwartzman ML Levere RD Kappas A Abraham NG Identification of binding sites for transcription factors NF-kappa B and AP-2 in the promoter region of the human heme oxygenase 1 gene Proc Natl Acad Sci USA 1994 91 5987 5991 10.1073/pnas.91.13.5987 8016102 PMC44122

[B48] Zhou LZ Johnson AP Rando TA NF-κB and AP-1 mediate transcriptional responses to oxidative stress in skeletal muscle cells Free Radic Biol Med 2001 31 1405 1416 10.1016/S0891-5849(01)00719-5 11728812

[B49] Maehara K Hasegawa T Isobe KI A NF-κB p65 subunit is indispensable for activating manganese superoxide: dismutase gene transcription mediated by tumor necrosis factor-α J Cell Biochem 2000 77 474 486 10.1002/(SICI)1097-4644(20000601)77:3<474::AID-JCB12>3.0.CO;2-H 10760955

[B50] Beetstra JB van Engelen JG Karels P van der Hoek HJ de Jong M Docter R Krenning EP Hennemann G Brouwer A Visser TJ Thyroxine and 3,3',5-triiodothyronine are glucuronidated in rat liver by different uridine diphosphate-glucuronyltransferases Endocrinol 1991 128 741 746 10.1210/endo-128-2-741 1899220

[B51] Luci S Kluge H Hirche F Eder K Clofibrate increases hepatic triiodothyronine (T3)- and thyroxine (T4)-glucuronosyltransferase activities and lowers plasma T3 and T4 concentrations in pigs Drug Metab Dispos 2006 34 1887 1892 10.1124/dmd.106.011379 16896063

[B52] Vansell NR Klaassen CD Effect of microsomal enzyme inducers on the biliary excretion of triiodothyronine (T(3)) and its metabolites Toxicol Sci 2002 65 184 191 10.1093/toxsci/65.2.184 11812922

[B53] Hood A Allen ML Liu Y Liu J Klaassen CD Induction of T(4) UDP-GT activity, serum thyroid stimulating hormone, and thyroid follicular cell proliferation in mice treated with microsomal enzyme inducers Toxicol Appl Pharmacol 2003 188 6 13 10.1016/S0041-008X(02)00071-6 12668117

[B54] Xu C Li CY Kong AN Induction of phase I, II and III drug metabolism/transport by xenobiotics Arch Pharm Res 2005 28 249 268 10.1007/BF02977789 15832810

[B55] Wang H LeCluyse EL Role of orphan nuclear receptors in the regulation of drug-metabolising enzymes Clin Pharmacokinet 2003 42 1331 1357 10.2165/00003088-200342150-00003 14674787

[B56] de Haan JB Nrf2 activators as attractive therapeutics for diabetic nephropathy Diabetes 2011 60 2683 2684 10.2337/db11-1072 22025774 PMC3198074

